# Comparative transcriptomics of sympatric species of coral reef fishes (genus: *Haemulon*)

**DOI:** 10.7717/peerj.6541

**Published:** 2019-03-01

**Authors:** Moisés A. Bernal, Groves B. Dixon, Mikhail V. Matz, Luiz A. Rocha

**Affiliations:** 1 Department of Biological Sciences, State University of New York at Buffalo, Buffalo, NY, USA; 2 Department of Integrative Biology, University of Texas at Austin, Austin, TX, USA; 3 Institute for Biodiversity, Science and Sustainability, California Academy of Sciences, San Francisco, CA, USA

**Keywords:** Gene expression, Speciation, TagSeq, Tropical Western Atlantic, Ichthyology, Divergent selection

## Abstract

**Background:**

Coral reefs are major hotspots of diversity for marine fishes, yet there is still ongoing debate on the mechanisms that promote divergence in these rich ecosystems. Our understanding of how diversity originates in this environment could be enhanced by investigating the evolutionary dynamics of closely related fishes with overlapping ranges. Here, we focus on grunts of the genus *Haemulon*, a group of coral reef fishes with 15 species in the Western Atlantic, 11 of which are syntopic.

**Methods:**

Wild fish samples from three sympatric species of the Caribbean: *Haemulon* *flavolineatum*, *H. carbonarium* and *H. macrostomum*, were collected while SCUBA diving. RNA was extracted from livers, and the transcriptomes were assembled and annotated to investigate positive selection (Pairwise *d*_N_/*d*_S_) and patterns of gene expression between the three species.

**Results:**

Pairwise *d*_N_/*d*_S_ analyses showed evidence of positive selection for genes associated with immune response, cranial morphology and formation of the anterior–posterior axis. Analyses of gene expression revealed that despite their sympatric distribution, *H. macrostomum* showed upregulation of oxidation-reduction machinery, while there was evidence for activation of immune response in *H. carbonarium*.

**Discussion:**

Overall, our analyses suggest closely related grunts show important differences in genes associated with body shape and feeding morphology, a result in-line with previous morphological studies in the group. Further, despite their overlapping distribution they interact with their environment in distinct fashions. This is the largest compendium of genomic information for grunts thus far, representing a valuable resource for future studies in this unique group of coral reef fishes.

## Introduction

Genetic studies on marine fishes have revolutionized our understanding of patterns of speciation and population connectivity, while providing basic information for the implementation of conservation strategies (Rocha, Craig & Bowen, 2007). However, most of these studies are based on a limited number of markers assumed to be neutral, leading to incomplete understanding of the dynamics of divergence between closely related taxa. The significant cost-reduction of massively parallel sequencing over the past decade has made it feasible to sequence large numbers of coding regions in non-model organisms (McCormack et al., 2013; Rocha et al., 2013). Thus, the assembly and annotation of the complete set of coding sequences of a particular organism, i.e. the transcriptome, is a convenient tool for studying their evolutionary history (Elmer et al., 2010; Limborg et al., 2012; Manousaki et al., 2013; Wang et al., 2015; Ma et al., 2016). Since divergence of closely related groups can be influenced by changes in coding regions, as well as those affected by differential gene expression, transcriptomes are well-suited tools for studying the genomic basis of adaptation and isolation of closely related marine fishes.

The scarcity of physical barriers in the ocean, as well as the potential for connectivity across broad distances during larval phases, represent important challenges for traditional views of speciation that require complete reproductive isolation to explain differentiation (i.e.: allopatry; Rocha & Bowen, 2008; Bowen et al., 2013). Mechanisms such as resource partitioning, sexual selection and habitat preferences could be especially important to explain the observed diversity of marine fishes associated with coral reefs, as these ecosystems provide a wide array of resources that can promote ecological differentiation among closely related lineages (Puebla, 2009). Hence, closely related coral reef fishes with broad sympatric ranges are convenient to understand the role resource partitioning and ecological preferences play on the divergence of marine organisms. A notable example in coral reef fishes is the genus *Haemulon*, which is composed of 21 nominal species distributed in the Tropical Western Atlantic (TWA; 15 species) and the Tropical Eastern Pacific (TEP; six species; Lindeman & Toxey, 2003; Robertson & Van Tassell, 2015). In both ocean basins, most of the sister species have completely overlapping ranges, with no apparent physical barriers separating them (Rocha et al., 2008; Tavera et al., 2012). Detailed information concerning the reproductive behavior of grunts is lacking, but their fecundity is highest between March and May (Shinozaki-Mendes et al., 2013), and anecdotal observations suggest they form large spawning aggregations for breeding (Domeier & Colin, 1997). In addition, previous studies have reported hybridization between closely related grunts with overlapping ranges (Rocha et al., 2008; Bernardi et al., 2013; Bernal et al., 2017).

**Figure 1:**
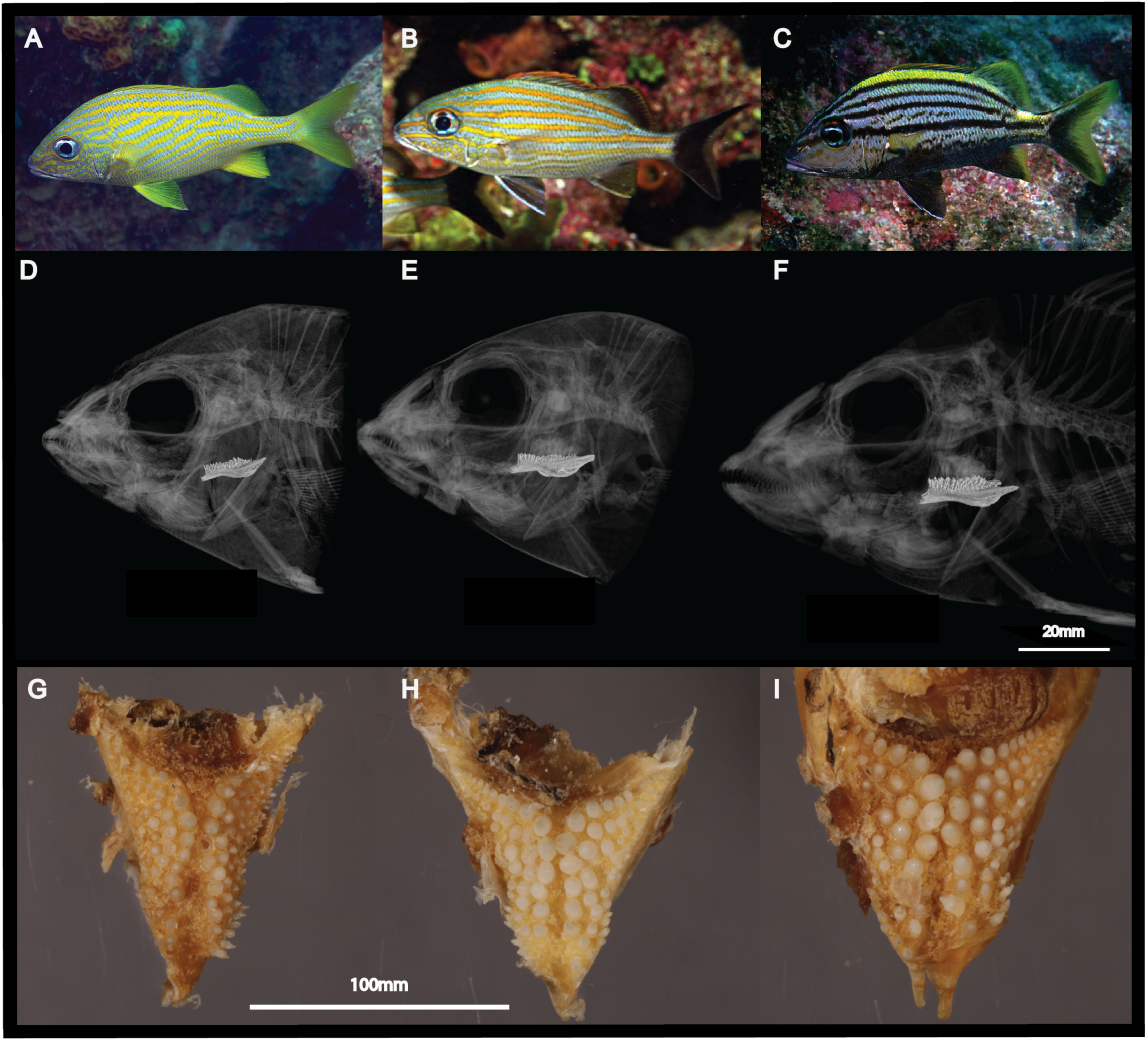
Morphological differences between the three sympatric species of Haemulon. Grunts are characterized by differences in their head and pharyngeal morphology, which are potentially associated with their feeding preferences. This figure shows photographs of H. flavolineatum (A), H. carbonarium (B) and H. macrostomum (C) in the wild; computed tomography scans of the cranium (D–F); and photographs of the lower pharyngeal teeth (G–I). Image credit: LA Rocha, EL Stanley, MA Bernal.

The present study focuses on three closely related species with overlapping distributions in the TWA: *Haemulon* *carbonarium* (Poey, 1860), *H. flavolineatum* (Desmarest, 1823) and *H. macrostomum* (Günther, 1859; Fig. 1). The phylogeny of the group suggests *H. carbonarium* and *H. macrostomum* are sister species, with *H. flavolineatum* being the closest relative to this lineage (Rocha et al., 2008; Tavera et al., 2012). These species are commonly seen schooling together in shallow reefs during the day, and move to sand flats and seagrass beds at night to feed on benthic invertebrates (Tulevech & Recksiek, 1994; Burke, 1995; Lindeman & Toxey, 2003). Coalescent estimates indicate the three species shared a most recent common ancestor approximately 5 Ma, while the split between *H. carbonarium* and *H. macrostomum* occurred approximately 2 Ma (Tavera et al., 2012; Tavera, Acero & Wainwright, 2018). Closely related grunts show considerable differences in diets (Randall, 1967; Pereira et al., 2015) and feeding behaviors (i.e. transitions from benthic to pelagic feeding; Tavera, Acero & Wainwright, 2018). For example, it has been suggested that *H. flavolineatum* feeds mostly on soft-bodied invertebrates and small crabs, while *H. carboraium* and *H. macrostomum* have more durophagous diets (Randall, 1967). Further, phylogenetic studies indicate that coral reef ecosystems accelerated the rate of morphological diversification of reef-associated haemulids, as there are considerable differences in feeding morphology between closely related species (Fig. 1; Price et al., 2013; Tavera, Acero & Wainwright, 2018). Because of these ecological differences, their recent origin and lack of population structure throughout their range (Purcell et al., 2006; Puebla, Bermingham & McMillan, 2012) it has been proposed that allopatric speciation followed by range expansion is an unlikely explanation for this diversification (Rocha et al., 2008). With this in mind, the genus *Haemulon* is a well-suited candidate for exploring the evolutionary mechanisms underlying ecological and morphological divergence of closely related marine fishes, using next-generation sequencing techniques.

In order to expand our understanding of the evolutionary dynamics of grunts, we sequenced, assembled and annotated liver transcriptomes of the sympatric species *H. carbonarium*, *H. flavolineatum* and *H. macrostomum*. In addition, we employed two different molecular approaches to characterize the differences between the three species: pairwise *d*_N_/*d*_S_ analyses to unravel genes showing evidence of positive selection, and analyses of liver gene expression that could provide insights into the ecological differences of the three species. This study represents the largest compendium of genomic information for the genus *Haemulon* to date, enhancing the resources available for the study of diversification of coral reef fishes.

## Materials and methods

### Specimen collections

Specimens of the three sympatric species of grunts were collected by SCUBA divers using pole spears in the Bocas del Toro Archipelago, Panama in March of 2012 (MiAmbiente Panama Permit SC/A-412). Individuals were euthanized by pithing immediately after collection (IACUC protocol AUP-2011-00172, University of Texas at Austin). In total, eight specimens of *H. carbonarium*, 17 of *H. flavolineatum* and 11 of *H. macrostomum* were collected in 2 days within the same 4-h period (9 am to 1 pm). Of the three species, *H. flavolineatum* was the most abundant and easiest to catch, while *H. carbonarium* and *H. macrostomum* were less common and warier. Dives were on average 60 min long, and liver samples were preserved in RNAlater (ThermoFisher) on the boat immediately after each immersion. Samples were initially stored at −20 °C freezers of the Smithsonian Tropical Research Institute Bocas del Toro Station, to be finally archived at −80 °C at the Center of Comparative Genomics of the California Academy of Sciences. Grunts lack sexual dimorphism, so we inspected the gonads of the collected individuals in order to determine their sex. The individuals collected did not show gonadal development, and sex could not be included as a factor for the analyses described below.

### Transcriptome sequencing and annotation

Total RNA was extracted from all liver samples using the RNAqueous Kit (Life Technologies, Carlsbad, CA, USA), following the manufacturer’s instructions. Final extractions were eluted in 40 µl of elution buffer, and treated with RNA-free-DNAase. The quantity and quality of the extractions were assessed with a Nanodrop spectrophotometer (Thermo Scientific, Waltham, MA, USA) and via electrophoresis in a 2% agarose gel. Extracted samples contained between 400 and 600 ng/µl of RNA.

The preparation of normalized cDNA libraries for transcriptome sequencing followed the protocol of Meyer et al. (2009), with modifications for Illumina sequencing. Only one sample of each species was used for the transcriptome library, which was chosen based on the best concentration and quality. First-strand cDNA synthesis was prepared with 500 ng of RNA. The libraries of the three individuals were sequenced as 100 bp paired end reads using an Illumina HiSeq 2000, at the Genomics Sequencing and Analysis Facility at the University of Texas at Austin. Preprocessing of samples was done following the Tag-Based RNAseq pipeline (https://github.com/z0on/tag-based_RNAseq). Removal of low-quality reads and PCR duplicates was done with *fastq_quality_filter* and *fastx_clipper*, respectively (FastX Tool Kit; Gordon & Hannon, 2010). Removal of duplicated reads was done with “*rnaseq_clipper.pl”*. The processed reads for the studied species are archived as BioProject PRJNA497743, in the Sequence Read Archive of the National Center for Biotechnology Information (NCBI).

The de novo assembly for each species was performed using Trinity V2.0.2 (Haas et al., 2013), generating contigs larger than 200 bp, with the default settings. The program CD-HIT (Fu et al., 2012) was used to identify potential isoforms. Transcripts were considered isoforms if they had more than 99% identity and at least 30% overlap. The assembled contigs were screened for contamination by vectors using the NCBI UniVec Database (accessed on August of 2014), and contigs producing matches with ≤1e^−10^ were removed. The annotation of the remaining contigs was based on UniProt and Trembl databases (downloaded on April, 2017; UniProt Consortium, 2014), using a stand-alone version of BLASTx (BLAST 2.2.28+). The e-value cutoff for matches leading to annotation was 1e^−5^. We also extracted the corresponding Gene Onthology annotation of each contig, using the script “*getGOfromUniProtKB.pl”*. Further, the assembled contigs were annotated with the Kyoto Encyclopedia of Genes and Genomes (KEGG; Kanehisa et al., 2016), using the online resource GhostKOALA (http://www.kegg.jp/ghostkoala/; Kanehisa et al., 2016). For this annotation, the amino-acid sequence of the longest isoform for each particular gene was extracted using the script “*fasta2SBH.pl*”, which resulted in 15,993 sequences for *H. carbonarium*, 14,571 for *H. flavolineatum* and 12,245 for H*. macrostomum*. The annotation was done against the KEGG gene database that retains KO content at the genus level for prokaryotes, and at the family level for eukaryotes. The assembled transcriptomes for each species, as well as their corresponding annotation to the different databases are presented in the Datasets S1–S3. The percentage of assembled contigs that aligned to 75% or more of the reference sequence (i.e.: contiguity) was estimated using the script “*contiguity.pl*”. Completeness of the transcriptome assembly was assessed using the Benchmarking Universal Single-Copy Orthologs (BUSCO) database for both vertebrates (2,586 orthologs) and ray-finned fishes (4,584 orthologs; Simão et al., 2015). The graph of completeness was generated with the BUSCO summary python scripts (available at: http://gitlab.com/ezlab/busco). Unless otherwise specified, all the scripts used for the transcriptome annotation and estimates of contiguity are available at: https://github.com/z0on/annotatingTranscriptomes.

### Analysis of orthologous sequences

Protein coding sequences were extracted from each of the transcriptomes based on BLASTx alignments to the Uniprot databases, using the script “*CDS_extractor_v2.pl*” (https://github.com/z0on/annotatingTranscriptomes). This script offers the advantage of traversing frameshifts within the matching region. An initial set of orthologs was obtained conducting a three-way Reciprocal Best BLASTp hits between the species’ extracted coding sequence (RBB; using the transcriptome of *H. macrostomum* as reference). A sequence was considered an ortholog if the reciprocal match had an e-value < 1e^−5^, more than 75% of sequence identity, and if the alignment length was >50% of the subject sequence. This initial candidate set of orthologous groups was quality controlled with two additional approaches. Following (Dixon, Bay & Matz, 2016), pair-wise *d*_*S*_ values among each set of candidate orthologous sequences were divided into three Gaussian components, based on Bayesian Information Criterion (BIC) implemented in *Mclust* (Fraley & Raftery, 2007). Sequences assigned to the third component (the 4% with the highest mean *d*_*S*_) were assumed to represent false positives and were removed from the analyses (Fig. S1). Finally, orthology was also assessed with the program FastOrtho using the default parameters (Wattam et al., 2013), and only ortholog assignments with concurrent calls from all methods were retained for downstream analyses. This final set of orthologs was composed of 84% of the initial genes of the RBB assessment.

**Figure 2:**
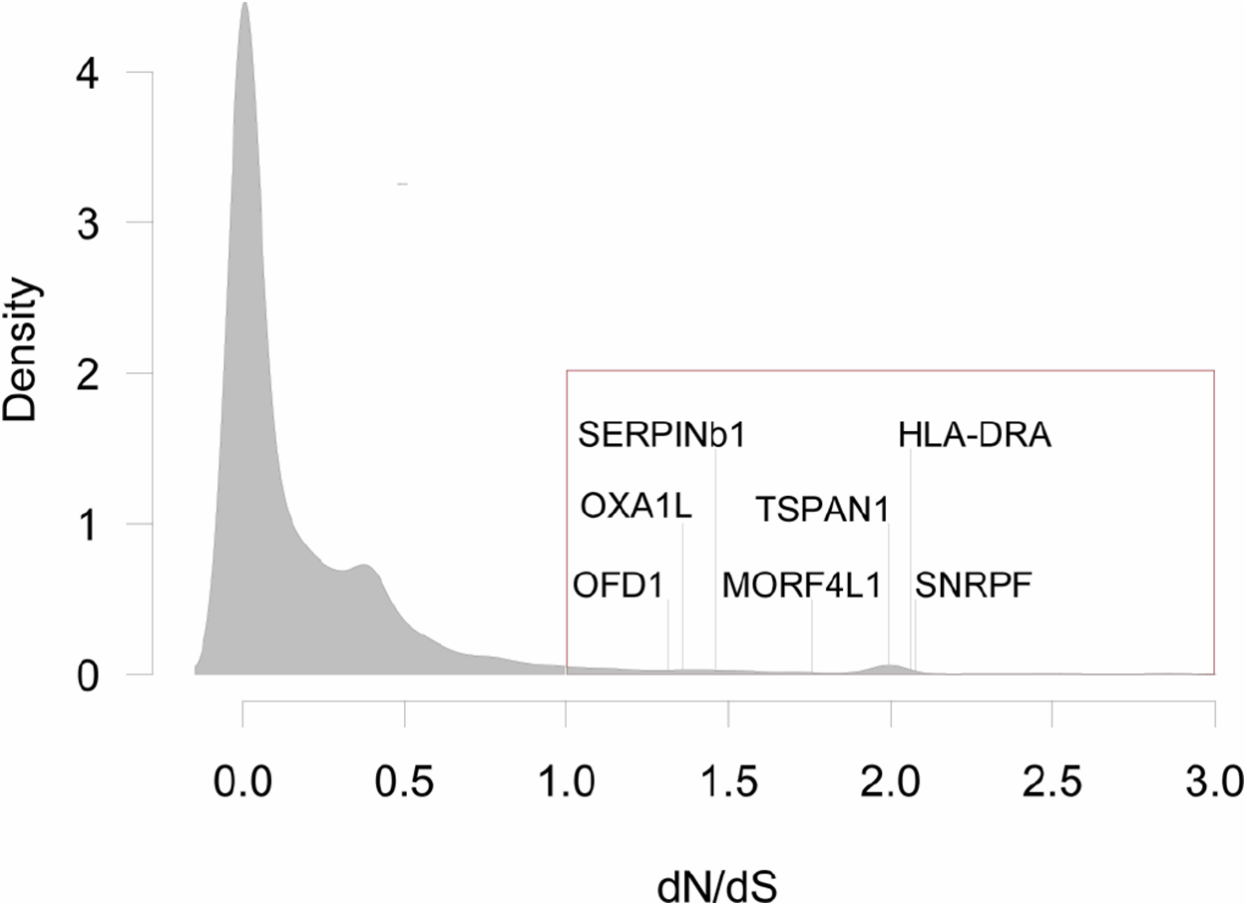
Distribution of *d*_N_/*d*_S_<3 values for all pair-wise comparisons between three species of grunts. Genes with *d*_N_/*d*_S_>1, are highlighted with the red box (*N* = 316, or 3.3% of all comparisons). There were seven genes with *d*_N_/*d*_S_>1 for all pair-wise comparisons: Oral-Facial-Digital Syndrome 1 Protein (OFD1), Mitochondrial Inner Membrane Protein (OXA1L), Leukocyte Elastase Inhibitor (SERPINb1), Mortality Factor 4-Like Protein 1 (MORF4L1), Tetraspanin 1 (TSPAN1), MHC Class II Antigen Beta Chain (HLA-DRA), and Small Nuclear Ribonucleoprotein F (SNRPF).

The pairwise *d*_N_/*d*_S_ ratios between orthologous genes of the three species were estimated with Codeml (Yang, 2007). This analysis follows the assumption that *d*_N_/*d*_S_ = 1 suggests orthologs are not under selection, *d*_N_/*d*_S_ < 1 indicates purifying selection and *d*_N_/*d*_S_ > 1 indicates positive selection (Nielsen, 2005). Upon examination of *d*_N_/*d*_S_ estimates for all ortholog pairs we found a small proportion of values that were unrealistically high, potentially due to remaining incorrectly assigned orthologs. To identify reasonable maximum ratios, we fitted Gausian mixture models to the pairwise *d*_N_/*d*_S_ indicative of positive selection (*d*_N_/*d*_S_ > 1). For each species, a four-component model was selected based on BIC and sequences assigned to the highest component were removed from the analysis. The transition between the highest and second highest component occurred at an average *d*_N_/*d*_S_ value of three, thus genes were only considered to be under positive selection if 1 < *d*_N_/*d*_S_ < 3 (Fig. 2; Fig. S2). One limitation of this analysis is that because it was based on one individual per species, we were not able to elucidate intra-specific differences. All scripts used for orthologs assignments are available at: https://github.com/grovesdixon/dNdS_for_grunts.

A gene ontology (GO) enrichment analysis was conducted with a Mann–Whitney U (MWU) test (Wright et al., 2015), for the *d*_N_/*d*_S_ values of the pairwise analyses. The test was performed with all *d*_N_/*d*_S_ values, yet here we only show the significantly enriched categories of the positively selected genes, that is genes 1 < *d*_N_/*d*_S_ < 3 (option “*g*” of the GO-MWU script). This assessment was done separately for the domains biological processes (BP), cellular components (CC) and molecular functions (MF). The false discovery rate (FDR) for GO terms was set to 10%. The scripts for the GO-MWU test are available at https://github.com/z0on/GO_MWU.

### Gene expression

To identify any potential ecological differences between the three sympatric species, TagSeq libraries were prepared for eight individuals of *H. carbonarium*, 17 of *H. flavolineatum* and 11 of *H. macrostomum*. Libraries were prepared following the protocol of Meyer, Aglyamova & Matz (2011) with modifications for introducing Illumina adapters. This protocol for library preparation produces a single tag per mRNA molecule at a random position near the 3′ end (Meyer et al., 2009). The first-strand cDNA synthesis used 300–500 ng of total RNA. After tagging each sample with an individual barcode, samples were size selected via gel-extraction, targeting fragments between 400 bp and 500 bp. Single end reads of 50 bp were sequenced in a single lane of Illumina HiSeq 2000 at the GASF.

Removal of low-quality reads and PCR duplicates was done with *fastq_quality_filter* and *fastx_clipper*, respectively (FastX Tool Kit; Gordon & Hannon, 2010). Adaptor trimming for the TagSeq reads was done with the script “*rnaseq_clipper.pl*” (https://github.com/z0on/tag-based_RNAseq). The reads for the gene expression analyses were archived in the Gene Expression Omnibus database of NCBI, under accession number GSE124093 (BioProject PRJNA497743). The annotated transcriptome of *H. macrostomum* was used as reference for mapping the TagSeq reads, as it had the lowest number of annotated transcripts (24,650 in total). Mapping to the reference transcriptome was done using Bowtie2 (Langmead & Salzberg, 2012), searching up to five matches per read. The number of reads that mapped to a particular transcript was extracted into a single text file using “*samcount_v.0.1.pl*”. As for the transcriptome analyses, unless specified, all the scripts used in this section are available at https://github.com/z0on/tag-based_RNAseq.

Differentially expressed genes (DEGs) were identified with DESeq2 (Love, Huber & Anders, 2014), using negative binomial generalized linear models. Normalization was done for all the gene counts using the three species as conditions. A likelihood ratio test (LRT) was used to determine the DEGs between the species, while excluding any genes differentially expressed within species (Love, Huber & Anders, 2014). For the principal coordinate analysis (PCoA), a matrix of normalized, log-transformed, nearly homoscedastic gene expression values was obtained via variance stabilizing transformation in DESeq2. The PCoA was performed with the R package *ape* (Paradis, Claude & Strimmer, 2004) based on Manhattan distances, which in this case correspond to the sum of log-fold differences across all genes. Pair-wise comparisons between the three species (*H. carbonarium* *vs* *H. flavolineatum*; *H. carbonarium* *vs* *H. macrostomum*; *H. macrostomum* *vs* *H. flavolineatum*) were conducted using the “Contrast” function of DESeq2, which determines whether the log2 fold change between two conditions is significantly different from zero (Love, Huber & Anders, 2014). A table with *p*-values and adjusted *p*-values of the log2 fold changes was exported and used for downstream analyses. A heat map with two-way hierarchical clustering was generated using the R package *pheatmap* (Kolde, 2015), using only the variance stabilized values of annotated significant genes of each of the pairwise comparisons.

A GO-MWU enrichment test (Wright et al., 2015) was done to determine if any GO terms were enriched based on the up- or down-regulated genes in the pairwise analyses. For this test, the unadjusted *p*-values from the pairwise comparisons of all genes were log-transformed. For downregulated genes, the value was kept negative while the upregulated log-*p* values were converted to positive (multiplying by −1). In this case the GO-MWU test determines enrichment of GO terms that correspond to upregulated or downregulated genes based on the list of all genes ranked according to the log-transformed unadjusted *p*-values. As in the *d*_N_/*d*_S_ GO-MWU analysis, the assessment was done separately for the domains BP, CC and MF, and FDR for GO terms was 10%.

## Results

### Transcriptome assembly and annotation

After removal of adaptors and quality control, 38,137,060 paired end reads were recovered for *H. carbonarium*, 38,999,126 for *H. flaviguttatum* and 48,779,031 for *H. macrostomum* (Table 1). The number of assembled isoform clusters after the Trinity assembly were 111,823 for *H. carbonarium*, 80,962 for *H. flavolineatum* and 81,132 for *H. macrostomum* (Table 1).

**Table 1:** Basic description of the transcriptome.

Species	Contigs	Max length (bp)	Average length (bp)	N50	Isogroups (CD-hit)	Annotated
HCAR	155,245	6,471	425	440	111,822	35,769
HFLAV	109,649	6,613	438	461	80,963	27,537
HMAC	111,768	6,079	443	465	81,132	24,650

**Note:**

Number of contigs, maximum length of contigs, average length, total length, N50 and number of isogroups for liver transcriptomes of *Haemulon carbonarium* (HCAR), *H. flavolineatum* (HFLA) and *H. macrostomum* (HMAC), after removal of potential contaminants. The minimum length of the contigs was set to 200 bp for the three species.

Using the UniProt and Trembl database, between 25% and 32.28% of the transcripts were annotated: 35,769 for *H. carbonarium* (15,784 genes), 27,537 for *H. flavolineatum* (13,497 genes) and 24,650 for *H. macrostomum* (12,802 genes). As expected, annotation success was proportional to contig size. For the three species, approximately 40% of the contigs were between 200 and 300 bp, while 5% were longer than 1,000 bp. The annotation with the KEGG database resulted in 11,755 annotated transcripts for *H. carbonarium* (73.50%), 10,751 for *H. flavolineatum* (73.80%) and 9,010 *H. macrostomum* (73.60%). The small size of the assembled contigs resulted in relatively low values of contiguity at 75% or more of the reference sequence: 12% for *H. carbonarium*, 13% for *H. flavolineatum* and 16% for *H. macrostomum*. Measures of completeness with respect to the vertebrate BUSCO dataset were 53.4% for *H. carbonarium*, 47.9% for *H. flavolineatum* and 46.6% for *H. macrostomum*. Completeness with the BUSCO ray-finned fish database was 31.9% for *H. carbonarium*, 29.6% for *H. flavolineatum*, and 30.3% for *H. macrostomum* (Fig. S3). It is important to highlight that these values of completeness could be influenced by the fact that the transcriptomes here presented are based on liver, and not a combination of tissues.

### Analysis of positive selection

The majority of ortholog calls made using RBB were concurrent with both the decomposition of synonymous mutations into Gaussian components and the analysis of FastOrtho (84%). The final set of high-confidence orthologs included 3,781 orthologous groups, that ranged in length from 150 bp to 3,216 bp. The results of the GO-MWU enrichment test are summarized on Table 2, while the list of all annotated genes under positive selection is available on Table S1. The majority of enriched GO terms for all comparisons were related to immune response and pathogen resistance, corresponding to the BP category. The pairwise comparisons between *H. carbonarium* and *H. flavolineatum* resulted in 74 genes with 1 < *d*_N_/*d*_S_ < 3, and the enrichment test resulted in five significant categories for BP, seven for CC and three for MF. Enriched categories under positive selection for this comparison included metabolism of carbohydrates and immune response (Fig. S4). Meanwhile, 116 genes showed evidence of positive selection in the comparison of *H. carbonarium* and *H. macrostomum*, including the developmental gene protein pumilio homolog 2 (PUM2; Table 2). The GO-MWU for these two species showed 37 significant categories for BP, nine for CC and 20 for MF (Fig. S5). Categories with significant enrichment in this comparison were related to immune response, lipid and carbohydrate metabolism and components of the cell membrane. Finally, the comparison between *H. flavolineatum* and *H. macrostomum* revealed 131 genes showing evidence of positive selection, the largest number among the three comparisons, including the gene cytokine stromal-cell derived factor 1 (SDF1). This comparison revealed enrichment of 25 categories for BP, six for CC and 20 for MF (Fig. S6), which included genes related with immune response, metabolism of lipids, carbohydrate binding and cargo receptors.

**Table 2:** Results for the analyses of positive selection.

Species	1 < *d*_N_/*d*_S_ < 3	Significant GO terms		
		BP	CC	MF
HCAR *vs* HFLA	72	**5-**Humoral immune response (21)	**5-**Transcriptionally active chromatin (6)	**3-**Scavenger receptor activity (8)
HCAR *vs* HMAC	116	**37-**TOR signaling (6)	**9-**Peroxisomal matrix (7)	**20-**Palmitoyl-CoA hydrolase activity(5)
HFLA *vs* HMAC	130	**25-**Long-chain fatty acid metabolic process (9)	**6-**External side of plasma membrane (18)	**12-**Scavenger receptor activity (8)

**Note:**

Number of orthologs under positive selection (1 < *d*_N_/*d*_S_ < 3) for pairwise comparisons between *H. carbonarium* (HCAR), *H. flavolineatum* (HFLA) and *H. macrostomum* (HMAC), along with the number of significantly enriched GO terms (in bold), the most significantly enriched GO term and the number of genes that belong to the most enriched GO term (in parenthesis).

In total, 50 orthologous groups showed evidence of positive selection in more than one comparison, including genes related with development of the nervous system (Protein Strawberry Notch homolog 2; PC4 And SFRS1-Interacting Protein; Monoacylglycerol Lipase ABHD12) and immunity (C-Type Lectin Domain Family 4-Member M; Growth Factor Receptor-Bound Protein 2; GTPase IMAP Family Member). Further, seven orthologs were under positive selection for all pairwise comparisons: Leukocyte Elastase Inhibitor (stress response), MHC Class II Antigen Alpha Chain (immunity), Mitochondrial Inner Membrane Protein OXA1L (metabolism), Mortality Factor 4-Like Protein 1 (stress response), Oral-Facial-Digital Syndrome 1 Protein (development), Small Nuclear Ribonucleoprotein F (replication) and Tetraspanin (immunity; Fig. 2).

### Differential gene expression

TagSeq reads of the three species mapped to 58,532 transcripts of *H. macrostomum,* 13,355 of which were annotated. The total number of reads that mapped to the transcriptome of *H. macrostomum* were: 18,043,592 for *H. carbonarium* (overall mapping efficiency 77.33%), 37,404,508 for *H. flavolineatum* (79.31%) and 28,098,252 for *H. macrostomum (*82.67%). The average number of counts per individual for the analysis of differential expression was 1,569,552 with a minimum of 578,011 and a maximum of 3,252,882.

**Figure 3:**
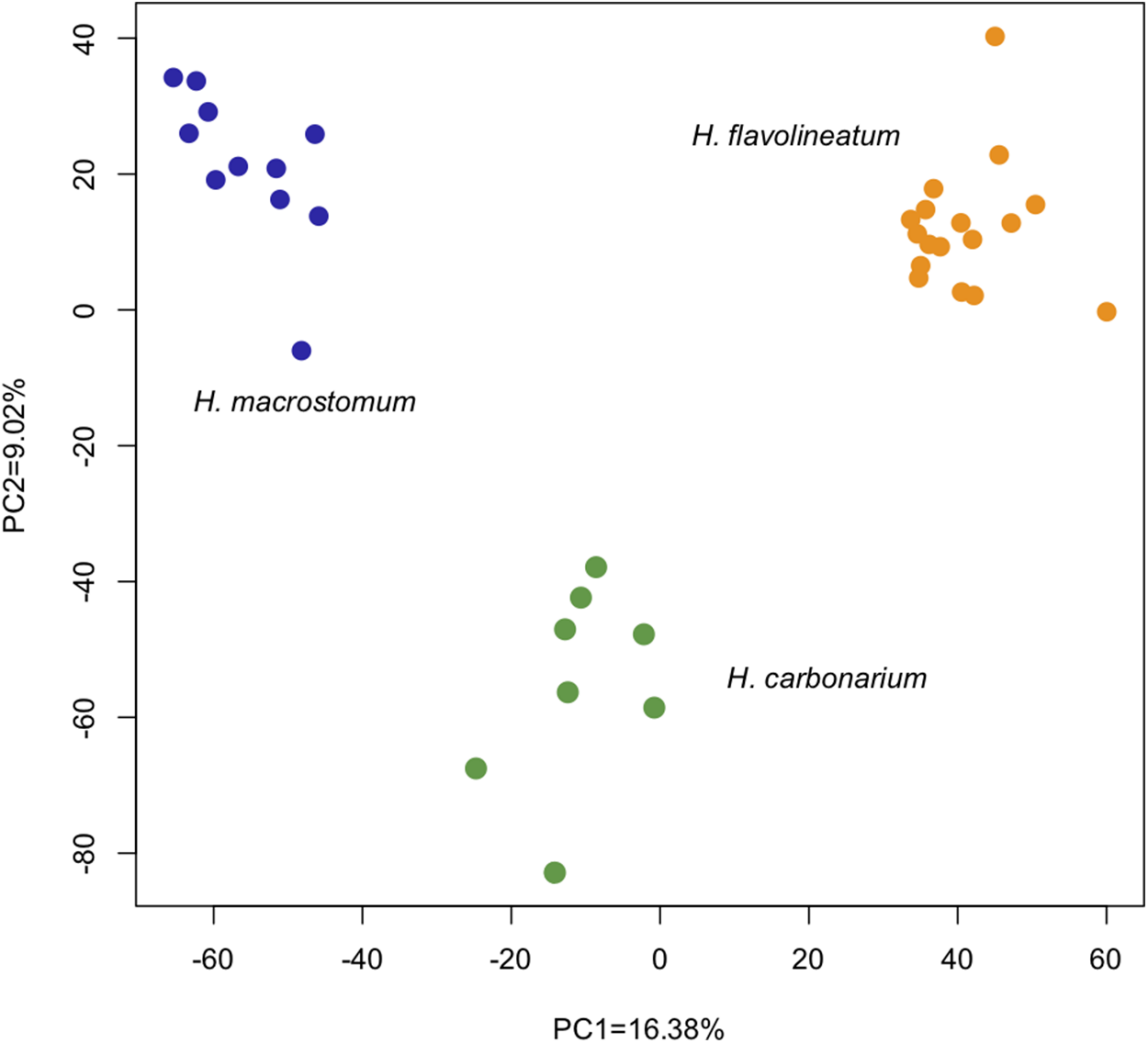
Principal Coordinate Analysis based on the read counts of differentially expressed genes between the three species of grunts based on the LRT. The first two axes captured 25% of the variance, and analyzed samples clustered together according to the species.

The analysis of differential expression with the LRT (Love, Huber & Anders, 2014) revealed 6,028 differentially expressed transcripts (1,855 annotated) between the three species (*p* < 0.01). The PCoA of all genes showed that despite inhabiting the same environment and being collected in a period of 2 days, individuals cluster tightly together by species. The first two principal components explained 25% of the variation: PC1 = 16.38% and PC2 = 9.02% (Fig. 3).

The first round of pairwise comparisons between species resulted 2,367 DEGs for the comparison of *H. carbonarium* and *H. flavolineatum*, 2,283 for *H. carbonarium* and *H. macrostomum*, and 4,427 between *H. flavolineatum* and *H. macrostomum*. However, after making the heatmaps for this initial round of contrasts, it was evident that half of the individuals of *H. flavolineatum* showed significant upregulation for Vitellogenin and Zona Pellucida genes (Fig. S7), suggesting that the individuals were female and preparing for reproduction.

**Figure 4:**
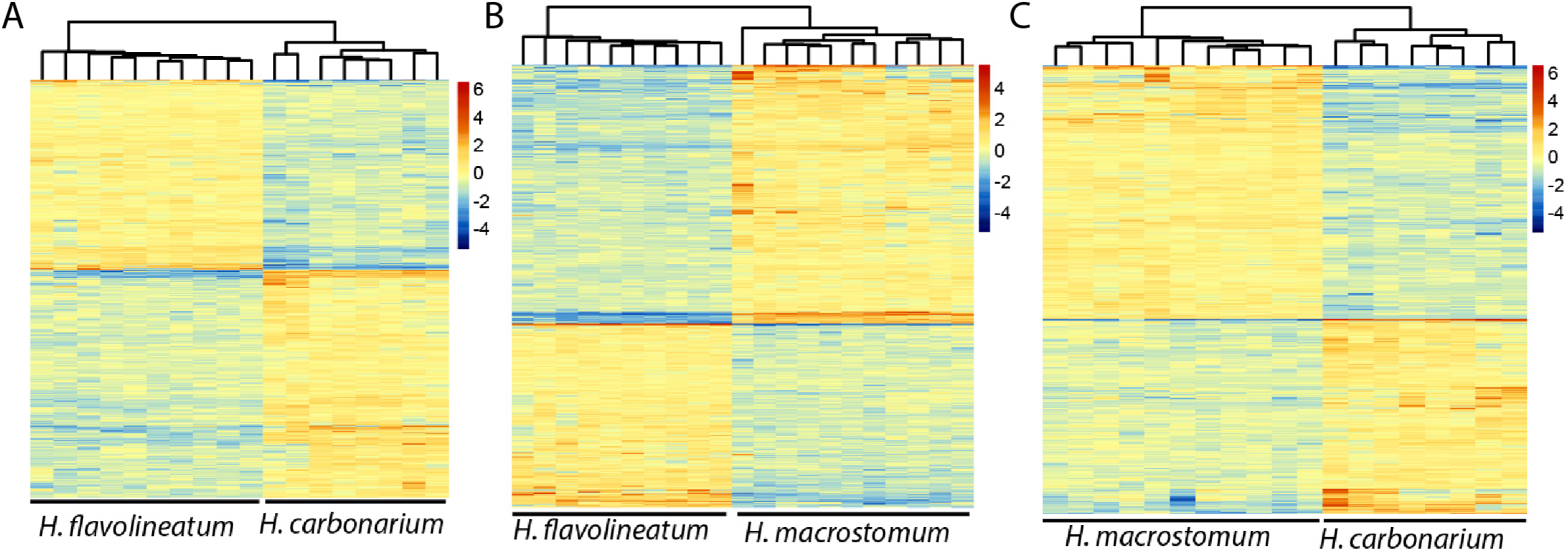
Heatmaps of the differentially expressed genes. Images represent the comparisons *Haemulon flavolineatum vs H. carbonarium* (A), *H. flavolineatum vs H. macrostomum* (B) and *H. carbonarium vs H. macrostomum* (C). Clustering of columns represent the grouping of samples by species. Samples of *H. flavolineatum* with upregulation for reproductive genes were excluded from this analysis.

In order to get a more accurate representation of contrasts between species, the test was repeated excluding the individuals of *H. flavolineatum* with highly upregulated reproductive genes, which resulted in fewer DEGs than initial estimates. Thus, we found 2,040 DEGs (677 annotated) in the comparison of *H. carbonarium* and *H. flavolineatum*, and the GO-MWU showed two terms enriched for BP, 13 for CC and five for MF (Table 3; Fig. S8). The comparison of *H. carbonarium* and *H. macrostomum* showed 2,283 DEGs (1,152 annotated), and this comparison had the highest amount of enriched terms for the GO-MWU analysis: 171 for BP, 57 for CC and 56 for MF (Table 3; Fig. S9). Finally, the comparison of *H. flavolineatum* and *H. macrostomum* showed 3,742 DEGs (1,355 annotated), revealing 22 enriched terms for BP, 28 for CC and 20 for MF (Table 3; Fig. S10). The heatmaps without the individuals with upregulated Vitellogenin are summarized in Fig. 4, while the complete lists of DEG for each comparison are presented in Table S2.

**Table 3:** Differentially expressed genes and GO terms enriched for the comparisons.

Species	Direction	DEG	Significant GO terms		
			BP	CC	MF
HCAR *vs* HFLA	Up	1,234	**1**-Immune response (177)	**10**-Microfibril (11)	**3**-Serine hydrolase activity (4)
	Down	806	**1**-Translation (182)	**3**-Cytosolic large ribos. Sub. (32)	**2**-Nutrient reservoir activity (7)
HCAR *vs* HMAC	Up	1,075	**36**-Asymmetric cell division (5)	**20**-Mit. respiratory chain II (8)	**30**-Eukaryotic translation init. (13)
	Down	1,208	**135**-rRNA base methylation (7)	**27**-Hemoglobin complex (6)	**26**-Retinol dehydrog. activity (5)
HFLA *vs* HMAC	Up	1,462	**7**-Ductus arteriosus closure (5)	**10**-Hemoglobin complex (6)	**6**-Nutrient reservoir activity (7)
	Down	2,280	**15**-Regulation of aminoacid transport (13)	**18**-Multi-eIF complex (6)	**14**-Protein kinase A regulatory sub (10)

**Note:**

Number of differentially expressed genes (DEG) that were up- and down-regulated in pairwise comparisons of *H. carbonarium* (HCAR), *H. flavolineatum* (HFLA) and *H. macrostomum* (HMAC), along with the number of significant GO terms enriched in the comparisons (in bold). Names represent the most significantly enriched GO term for the categories, and the numbers in parenthesis represent the genes that belong to the most enriched GO term.

## Discussion

Closely related fishes with completely overlapping distributions offer a great opportunity to study the mechanisms of divergence in coral reef ecosystems. Since there is an apparent paucity of physical barriers that could promote allopatric speciation in the sea, it is possible that a significant portion of the rich diversity of coral reef fishes originated as a result of exploiting the surrounding environments in different ways (Rocha & Bowen, 2008; Puebla, 2009; Bowen et al., 2013). With this in mind, we compared the transcriptome of three sympatric species of *Haemulon* with completely overlapping distributions in the TWA.

### Gene expression of wild fish samples

Originally, the analyses of liver gene expression were done to evaluate potential differences in diet between the three sympatric species, which have been previously reported in the literature (Randall, 1967; Pereira et al., 2015). However, we did not recover clear patterns of gene expression that could be unequivocally related to differences of feeding habits. Signatures of gene expression in liver can change based on metabolic conditioning (Bernal et al., 2018), life stage (Sarropoulou et al., 2016), temperature variation (Shama, 2015), salinity (Maryoung et al., 2015), among many other variables that were not controlled in this study. Since all the samples from this study were collected in the wild, it is challenging to assess if any specific conditions experienced by the fish throughout development could be responsible for the observed variation in expression. Hence, results corresponding to this section should be interpreted with caution.

A good example of the challenges and limitations of working with wild samples is *H. flavolineatum,* where half of the samples showed upregulation for Vitellogenin. This carrier protein is synthetized in the liver when fishes are getting ready to develop their oocytes (Mommsen & Walsh, 1988). Lipids, carbohydrates and phosphates are carried by Vitellogenin into the oocyte, and these compounds eventually become the egg yolk as maturation progresses (Mommsen & Walsh, 1988). With this in mind, it is likely that the collected individuals were at different stages of oocyte production. However, it is also possible that the individuals analyzed were of different sexes. Grunts lack the sexual dimorphism that characterizes many other coral reef fishes, and the collected samples did not show any signs of gonadal maturation to discern the gender. Removing the individuals that showed upregulation for vitellogenin from the pairwise comparisons resulted in a smaller number of DEG between *H. flavolineatum* and the sister species *H. carbonarium* and *H. macrostomum*. This finding highlights the importance of controlling for gonadal development in studies of liver gene expression in coral reef fishes, in order to avoid overestimation of DEG.

Interestingly, a considerable number of GO categories associated with mitochondrial function, respiratory chain, electron transport and oxidoreductase activity were upregulated for *H. macrostomum*, in comparison with *H. carbonarium* and *H. flavolineatum*. Upregulation of genes associated with these categories is usually associated with an increase of oxygen consumption, as a result of elevated metabolic activity (Zorov, Juhaszova & Sollott, 2014). It would be tempting to suggest that this pattern is caused by the stress of the collections; however, all the specimens in the study were collected with pole spears around the same window of time in a period of 2 days (from 9 am to 1 pm), and upregulation of the same pathways was not observed in the other two species. This observation could be related to the ecology of *H. macrostomum*, as most of the individuals of this species were collected on outer reefs exposed to more intense currents and wave action than the other two species. This pattern is consistent with previous underwater surveys of the Bocas del Toro area, where *H. macrostomum* is more common in exposed areas, while *H. flavolineatum* and *H. carbonarium* are generally seen in bays and in-shore reefs (Dominici-Arosemena & Wolff, 2005). This observation should be interpreted with caution, as comprehensive surveys, respirometry assays and behavioral observations are necessary to confirm this pattern of gene expression.

### Selection and differential expression of immunity genes

Pathogens and parasites have the ability to reduce fitness, which leads to intense selective pressure on genes related with immune response (Barreiro & Quintana-Murci, 2010; Phillips et al., 2018). Our analyses of pairwise *d*_N_/*d*_S_ showed positive selection for multiple genes related with immunity, which in turn resulted in significant enrichment of their corresponding GO terms. Some of the most notable examples included positive selection for Toll-like receptors, which encode for recognition of conserved sites of pathogens (Roach et al., 2005); Protein NLRC3, which modulate the activation of T-cells (Paria et al., 2016); E3 ubiquitin-protein ligase TRIM21, associated with anti-viral response (van der Aa et al., 2012); Lysozymes C and G, enzymes that can degrade bacterial cells walls during infection (Jiménez-Cantizano et al., 2008); and IL2 inducible T-Cell Kinase, which regulate the action of T-cells (Krasnov et al., 2013). The analyses also suggest positive selection for MHC Class I Alpha Chain and MHC Class II Alpha Chain, which encode for receptors on the membrane of immune cells, starting the immune response to potential pathogens in vertebrates. Studies suggest that selection in the MHC complex is much more intense than any other immunity-associated gene across the vertebrate tree, and it can even be influenced by sexual selection (i.e.: MHC dissasortative mating; Bernatchez & Landry, 2003; Ejsmond, Radwan & Wilson, 2014). These results indicate that our *d*_N_/*d*_S_ analysis was sensitive enough to detect genes commonly known to be under divergent selection, and that the three sympatric species of *Haemulon* appear to be responding to local pathogens in distinct fashions.

Interestingly, there was a pattern of upregulation of genes related with immune response for *H. carbonarium* with respect to its sister species *H. macrostomum*. The GO enrichment analysis for gene expression showed upregulated categories such as Response to Virus, Immune Response, Regulation of Lymphocyte Activation, Regulation of Cell Activation and Major Histocompatibility Complex. Further, there was downregulation of categories associated with ribosomal activity electron transport, which are signals of diminished growth and overall metabolic stress. Hence, it is possible that individuals of *H. carbonarium* were exposed to pathogens or viral infection during the time of the collection. This would imply that despite the similarity in habitat and the phylogenetic proximity, the two species could have different susceptibility to pathogens in their natural habitat. However, this observation would need further evaluation, as the number of individuals collected for this study was very limited considering their broad geographic distribution.

### Morphological differences and selection of developmental genes

Body plans of all fishes are controlled by a tightly regulated set of genes that vary in sequence, as well as in timing of expression, across different groups. Our results indicate that many of the genes with evidence for positive selection between the studied species are related to fundamental developmental processes in fish. For example, cytokine stromal-cell derived factor 1 (evidence of positive selection between *H. flavolineatum* and *H. macrostomum*), controls the migration of germ cells that express the chemokine CXCR4, promoting the appropriate formation of the endoderm, and later on, the formation of fast muscle fibers in fishes (Chong et al., 2007). This gene is also associated with the development of the lateral line, as expression on the side of the body defines the location of neuromasts in zebrafish embryos (David et al., 2002). The interactions between CXCR4/SDF1 are also important for the development of the forebrain of fishes, specifically the neurons related to the olfactory bulbs (Palevitch et al., 2010). Meanwhile, the RNA-binding protein pumilio homolog 2 (evidence of positive selection between *H. carbonarium* and *H. macrostomum*) is an RNA-binding protein expressed in the brain and gonads of rainbow trout (Kurisaki et al., 2007), as well as gonads, liver, kidney and brain of zebrafish (Wang et al., 2012). A study in zebrafish suggests this gene is essential for the development of the anterior-posterior axis during embryogenesis and plays a role in the formation of the nervous system (Wang et al., 2012). All comparisons suggested positive selection for the oral facial digital syndrome 1 (OFD1) and the PC4 and SFRS1 interacting protein 1 (PSIP1 or LEDGF). OFD1 has been extensively studied in humans, as mutations of this gene can lead to dysmorphia of the cranium, extremities and oral cavity, which often result in prenatal death (Prattichizzo et al., 2008). This protein is associated with centrosomes and primary cilia of vertebrates, and plays a vital role in tissue development and lateralization of organs (Romio et al., 2003; Prattichizzo et al., 2008). Changes in expression of OFD1 in zebrafish during embryogenesis leads to bent body axes, randomization of laterality, improper development of organs (including brain and heart) and deformity of jaws (Ferrante et al., 2008). Meanwhile, PSIP1 is a regulator of expression of *Hoxa* and *Hoxd* genes in mammals, genes that are key for the proper development of the anterior/posterior axis (Pradeepa et al., 2014). Mutations of the latter can lead to deformations of the cranium and torso of mice embryos (Sutherland et al., 2006).

Without extensive in situ hybridizations and gene manipulation experiments it would be impossible to pinpoint the precise role of the genes under positive selection in the divergence of the studied species. However, if we put our results in the context of previous studies of the Haemulidae family, it is plausible that positive selection of these coding regions is associated with the stark morphological differences between the three focal species, including body depth, head shape and pharyngeal morphology (Fig. 1; Lindeman & Toxey, 2003; Tavera, Acero & Wainwright, 2018). The morphological studies on haemulids suggest large morphological differences between sympatric sister species are common for grunts that inhabit coral reef ecosystems, especially for traits related with feeding morphology and prey capture (Price et al., 2013; Tavera, Acero & Wainwright, 2018). Further, differences in diet between closely related species have also led to differences in body shape, as at least a dozen transitions from benthic to pelagic feeding have been documented in haemulids (Tavera, Acero & Wainwright, 2018). Thus, it is hypothesized that large morphological differences of haemulids are promoted by character displacement and resource partitioning between closely related species, which is feasible thanks to the high diversity of prey items in coral reef ecosystems (Price et al., 2013). This could explain, at least in part, positive selection of genes associated to body shape between the closely related species analyzed in this study.

## Conclusions

Overall, the comparisons of grunts’ transcriptomes suggest that despite their close phylogenetic relationship and overlapping distributions, these species interact with their environment in very different ways, as they show considerable differences in metabolic and immunological processes. In addition, by finding positive selection of genes associated with feeding morphology and body shape, our results are in-line with hypotheses that suggest diversification of grunts has been promoted by resource partitioning between closely related groups. The sequencing, assembly and annotation of the transcriptome of these three species represents the most complete genomic resource for grunts to date, and will be an essential tool for future studies of this interesting group of coral reef fishes.

## Supplemental Information

10.7717/peerj.6541/supp-1 Supplemental Information 1 Supplementary Figures. PDF containing the Supplementary Figs. S1 through S10.

10.7717/peerj.6541/supp-2 Supplemental Information 2 Genes under positive selection for the pairwise dN/dS comparisons.

10.7717/peerj.6541/supp-3 Supplemental Information 3 Differentially expressed genes between the three sympatric species of grunts.

10.7717/peerj.6541/supp-4 Supplemental Information 4 Assembly and annotation files for *Haemulon flavolineatum*.

10.7717/peerj.6541/supp-5 Supplemental Information 5 Assembly and annotation files for *Haemulon carbonarium*.

10.7717/peerj.6541/supp-6 Supplemental Information 6 Assembly and annotation files for *Haemulon macrostomum*.

## Additional Information and Declarations

### Field Study Permissions

The following information was supplied relating to field study approvals (i.e., approving body and any reference numbers): MiAmbiente Panama provided permit number SC/A-412 for this study.

### DNA Deposition

The following information was supplied regarding the deposition of DNA sequences: The raw sequences for this project were are available at NCBI Bioproject PRJNA497743. CT scans for Haemulon are available at MorphoSource, media files: 66603 (https://www.morphosource.org/Detail/MediaDetail/Show/media_id/36283), 66606 (https://www.morphosource.org/Detail/MediaDetail/Show/media_id/36284), 66609 (https://www.morphosource.org/Detail/MediaDetail/Show/media_id/36285).

### Data Availability

The following information was supplied regarding data availability: Pipeline for the assembly and annotation of sequences: https://github.com/z0on/tag-based_RNAseq Pipeline for the analyses of dN/dS: https://github.com/grovesdixon/dNdS_for_grunts
