# Supplementary material for: Comparative transcriptomics of sympatric species of coral reef fishes (genus: Haemulon)
Source: PeerJ. 2019 Mar 1;7:e6541. doi: 10.7717/peerj.6541 (PMC6398375; doi:10.7717/peerj.6541)

SUPPLEMENTAL INFORMATION

Figure S1 Density curves of synonymous mutations to assess false orthologs for the pairwise comparisons of *H. carbonarium* (A), *H. flavolineatum* (B) and *H. macrostomum* (C). Histograms indicate overall distribution of pair-wise *dS* values for each pairwise comparison, and bars indicate the density of genes assigned to each Gaussian component (Component1= blue; 2= red; 3= green; all genes= black). Genes assigned to the third component (green) were excluded from the analyses, as they had the highest *dS* values and were assumed to be inaccurate calls.

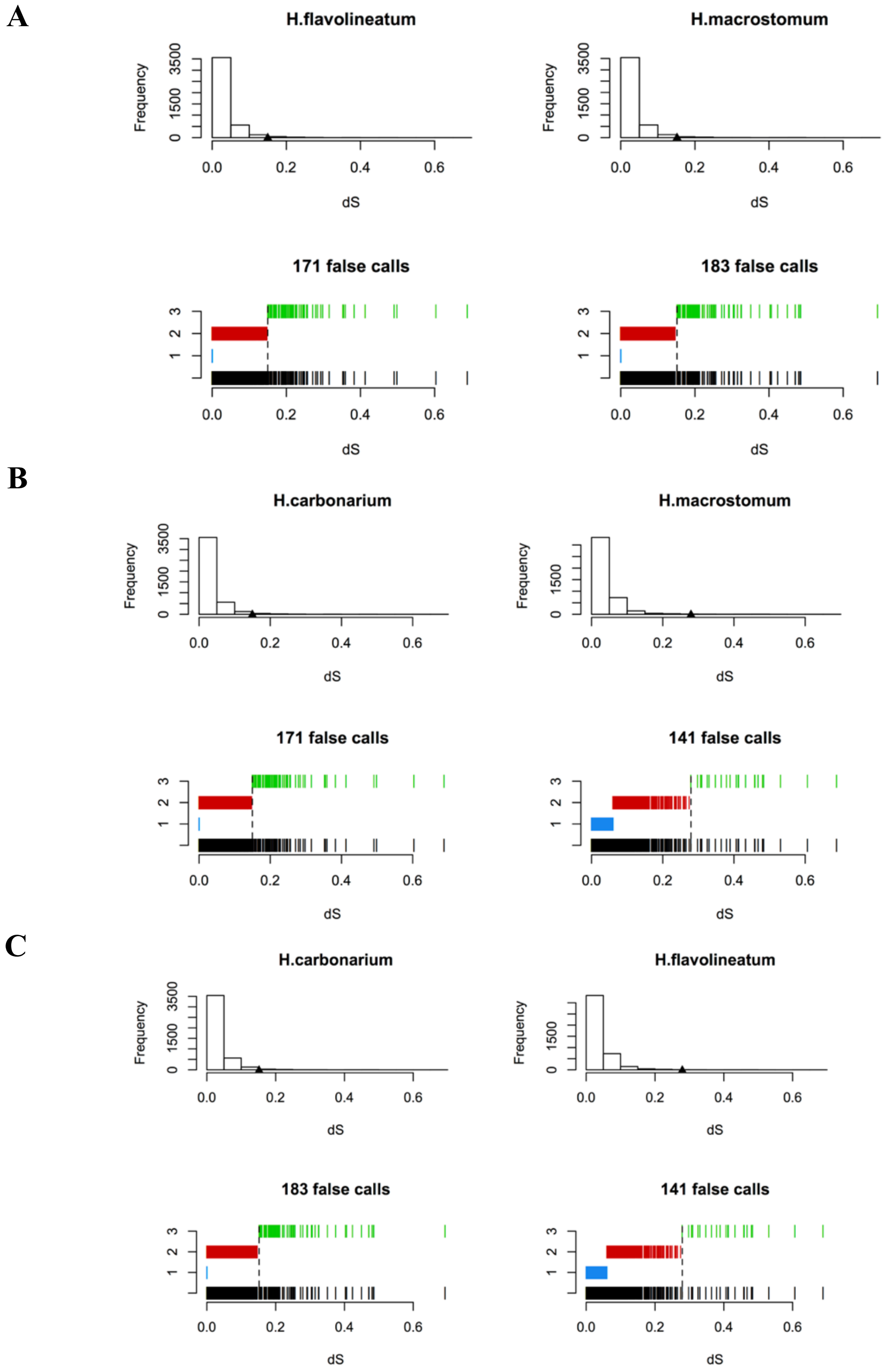

Figure S2 Histograms show the distribution of pairwise 1.0<dN/dS<4.0 for *H. carbonarium* (A), *H. flavolineatum* (B), *H. macrostomum* (C). Colored lines represent the density of four-component mixture models fitted to all *dN/dS* (between 0 and 20). The fourth component (teal) was assumed to capture false ortholog calls and was removed. For all three species, the transition between the third and fourth component (vertical black dotted line) occurred at a *dN/dS* value of 3.

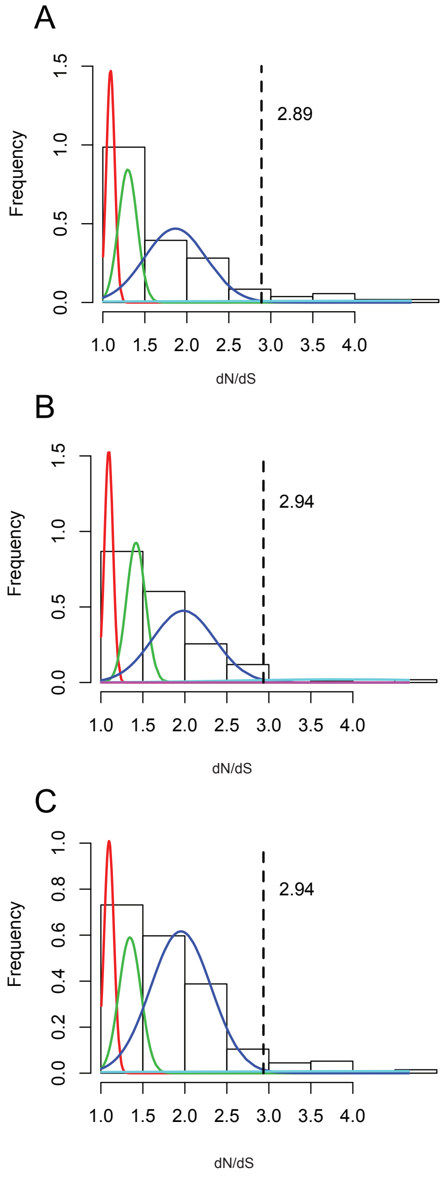

Figure S3 Bar graphs reflecting the completeness of the transcriptome in reference to the BUSCO ortholog database for vertebrates (A) and ray-finned fish (B) for the species *H. carbonarium* (HCAR), *H. flavolineatum* (HFLA) and *H. macrostomum* (HMAC).

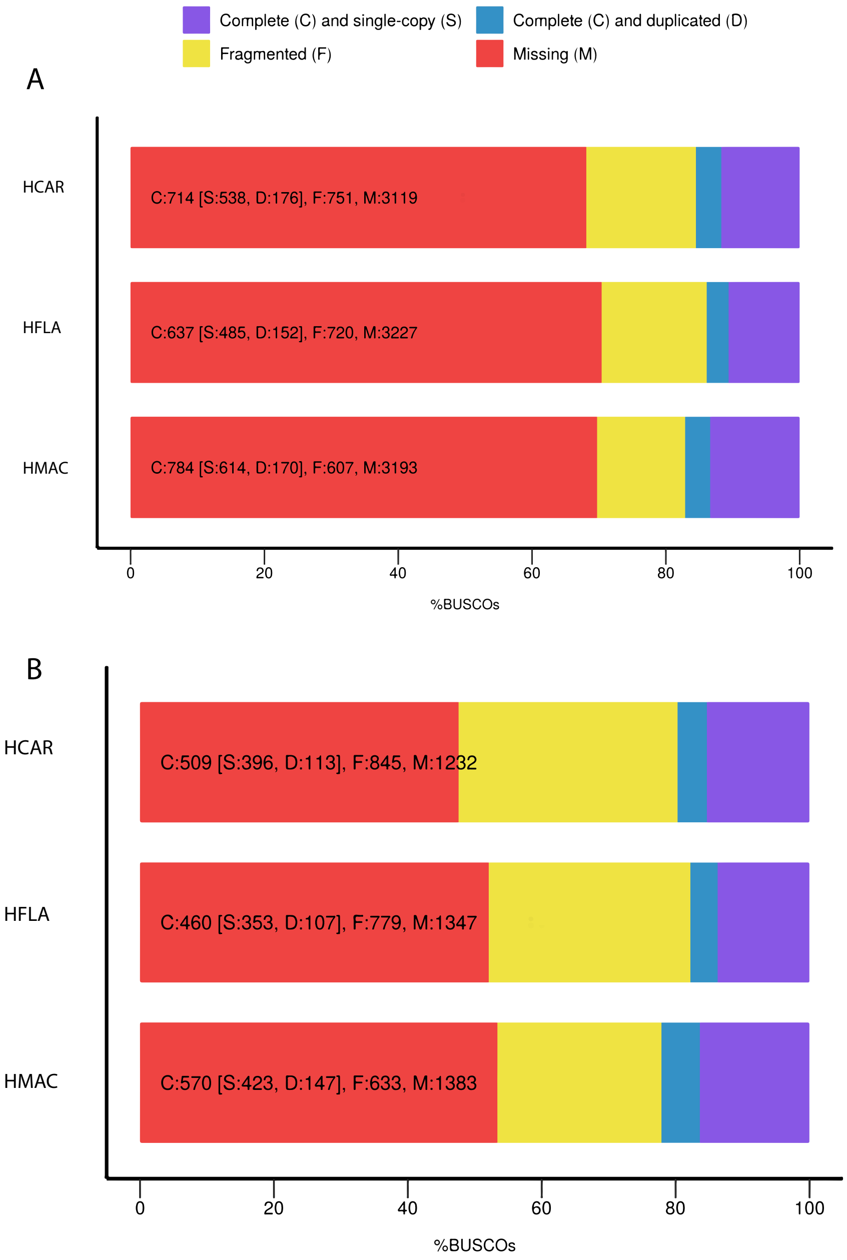

Figure S4 Gene Ontology (GO) terms significantly enriched for the d_N/d_S comparisons between *H. carbonarium* and *H. flavolineatum*, for the categories Biological Process (A), Cellular Component (B) and Molecular Function (C). These results include only genes under positive selection, and size and brightness of GO terms depends on the level of significance. Numbers that precede the category name are the genes present in this particular analysis over the total number of genes that belong to that category.

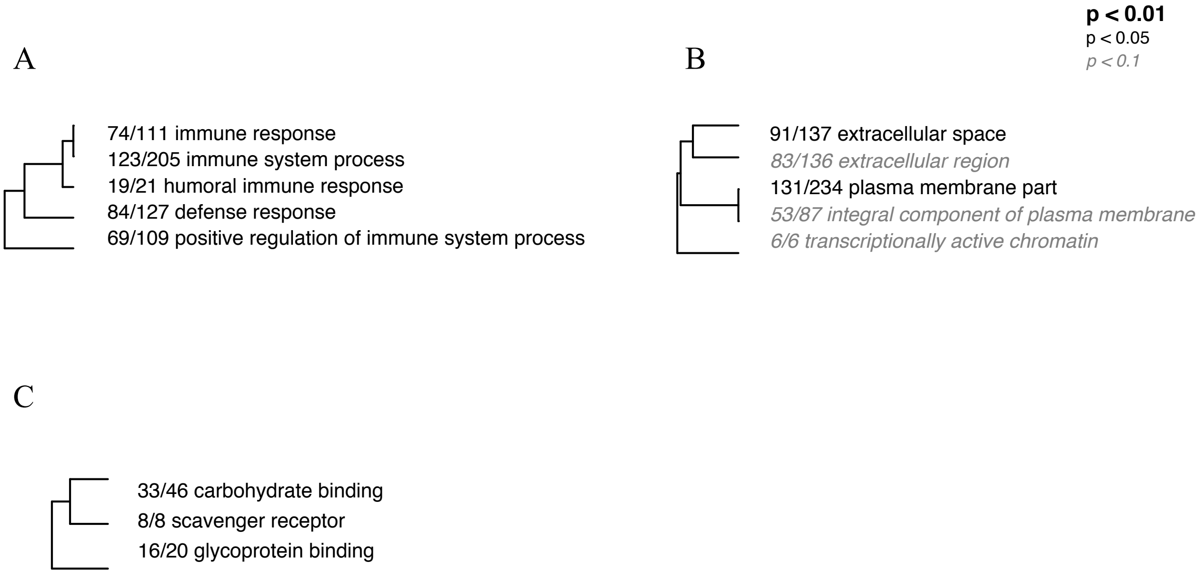

Figure S5 Gene Ontology (GO) terms significantly enriched for the d_N/d_S comparisons between *H. carbonarium* and *H. macrostomum*, for the categories Biological Process (A), Cellular Component (B) and Molecular Function (C). These results include only genes under positive selection, and size and brightness of GO terms depends on the level of significance. Numbers that precede the category name are the genes present in this particular analysis over the total number of genes that belong to that category.

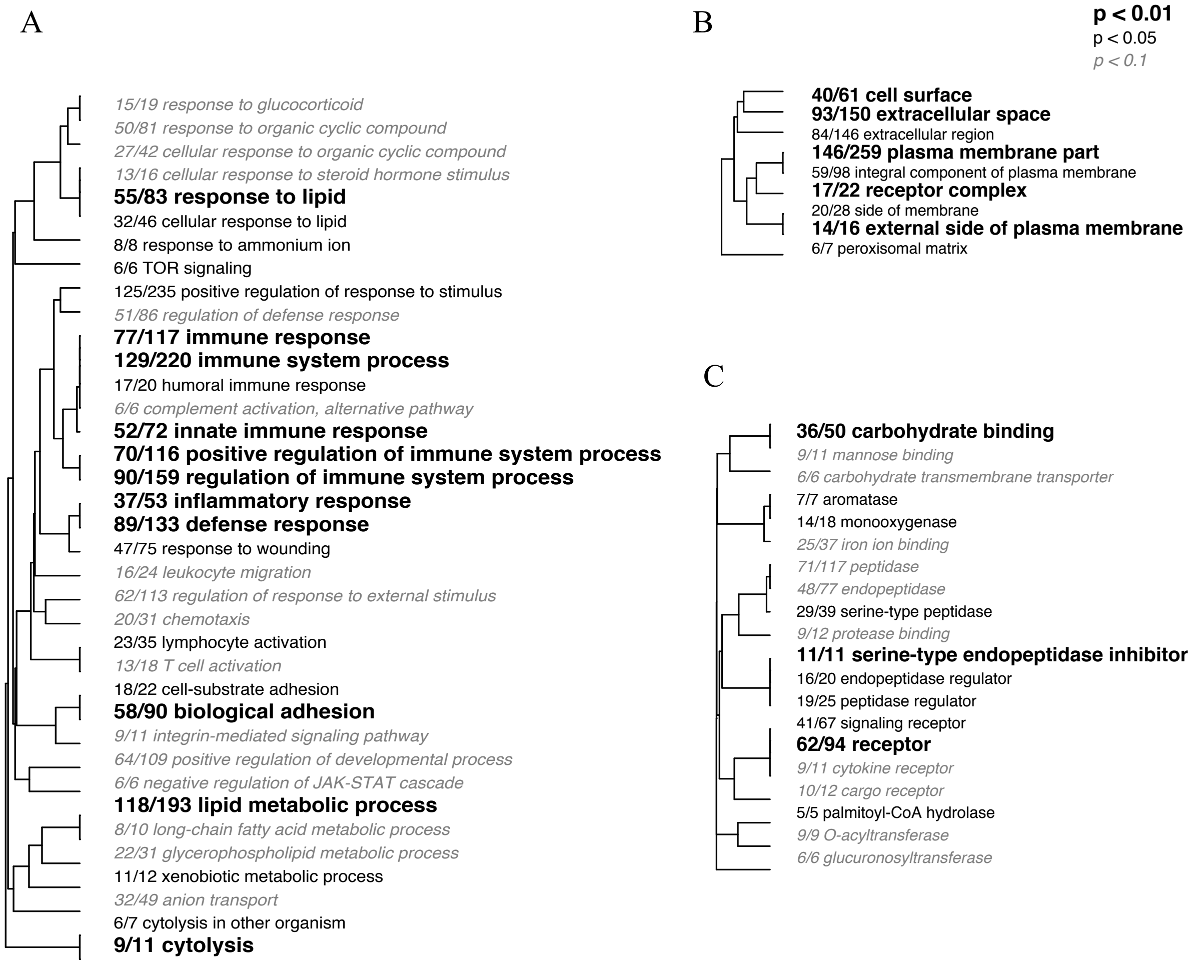

Figure S6 Gene Ontology (GO) terms significantly enriched for the d_N/d_S comparisons between *H. flavolineatum* and *H. macrostomum*, for the categories Biological Process (A), Cellular Component (B) and Molecular Function (C). These results include only genes under positive selection, and size and brightness of GO terms depends on the level of significance. Numbers that precede the category name are the genes present in this particular analysis over the total number of genes that belong to that category.

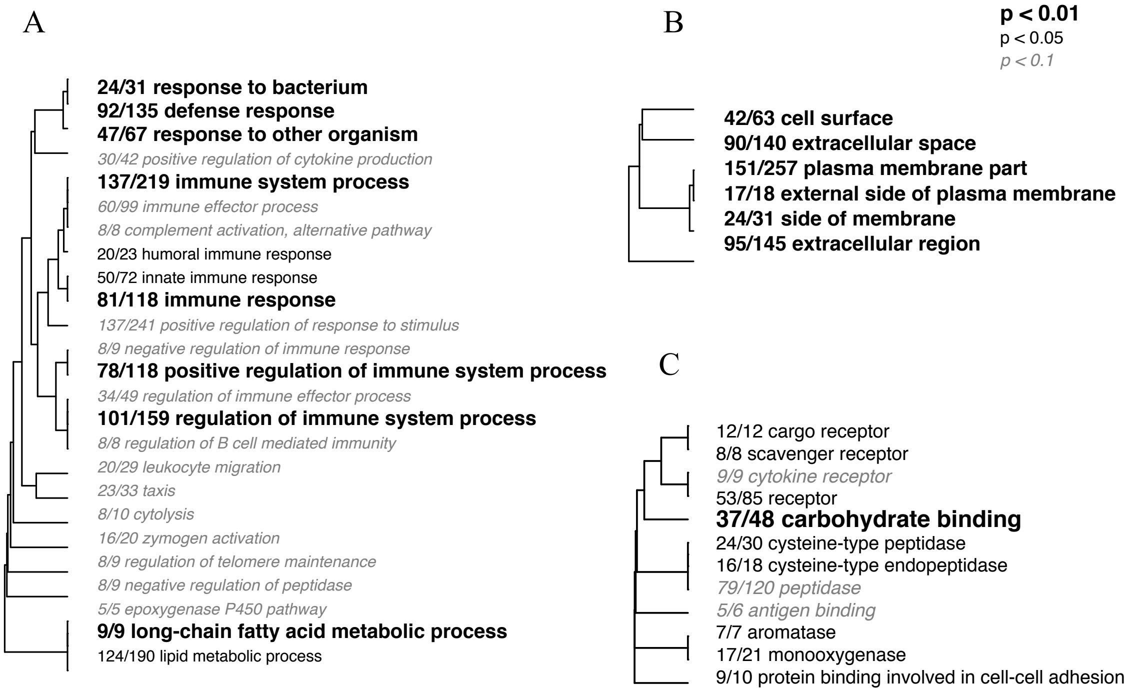

Figure S7 Example of a heatmap for transcripts of Vitellogenin and Zona Pellucida genes, which were highly upregulated for seven individuals of *Haemulon flavolineatum* (HFL; in bold). These individuals were removed from the analyses in order to get more accurate measures of differential expression and GO enrichment. The other individuals of *H. flavolineatum* have similar levels of expression to all the collected individuals of *H. macrostomum* (HMCR).

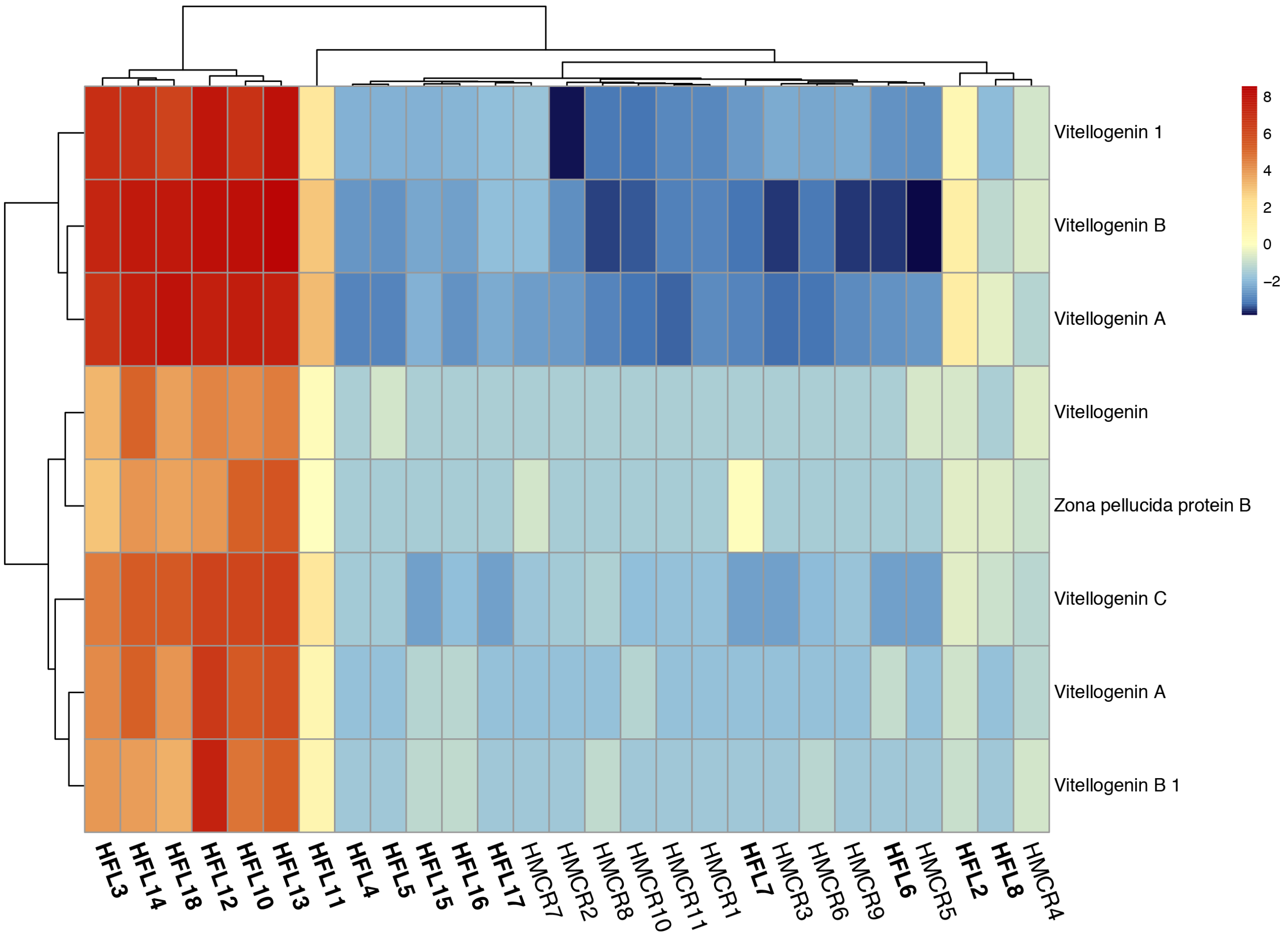

Figure S8 Gene Ontology (GO) terms significantly enriched in the comparison of gene expression of *H. carbonarium* and *H. flavolineatum*, for the categories Cellular Component (A) and Molecular Function (B). Size and brightness of GO terms depends on the level of significance, and numbers that precede the category name are the genes present in this particular analysis over the total number of genes that belong to that category. GO terms in red are upregulated categories for *H. carbonarium*, while blue ones are downregulated. The category of Biological Process only had two significant terms: Translation (downregulated, 182 sequences) and Immune Responses (upregulated, 177 sequences).

A

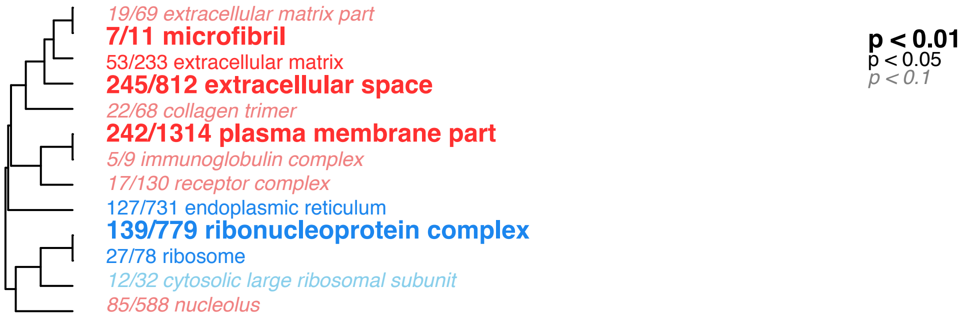

B

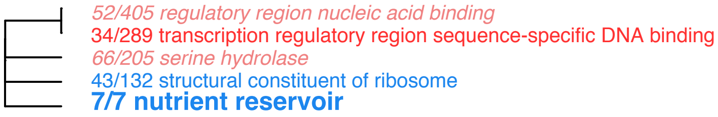

Figure S9 Gene Ontology (GO) terms significantly enriched with the MWU Test of gene expression for *H. carbonarium* and *H. macrostomum*, for the categories Biological Process (A), Cellular Component (B) and Molecular Function (C). Due to the large number of significant GO terms for BP (171), only those with a FDR<0.02 were included in the tree. Size and brightness of GO terms depends on the level of significance, and numbers that precede the category name are the genes present in this particular analysis over the total number of genes that belong to that category. GO terms in red are upregulated categories for *H. carbonarium*, while blue ones are downregulated.

A

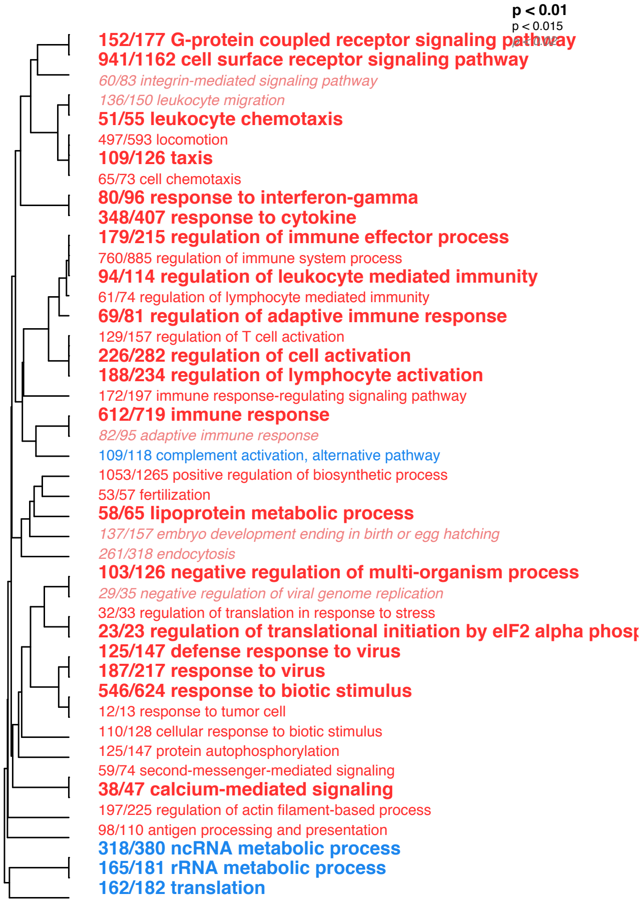

B

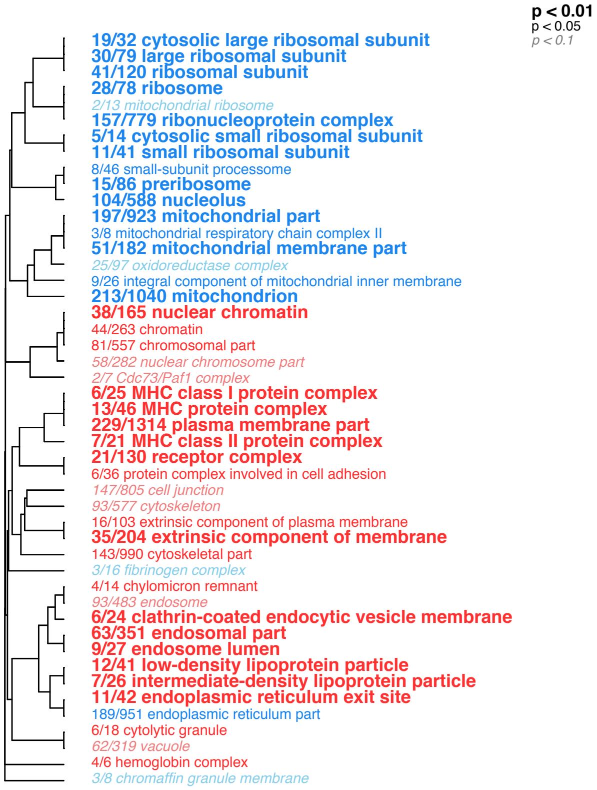

C

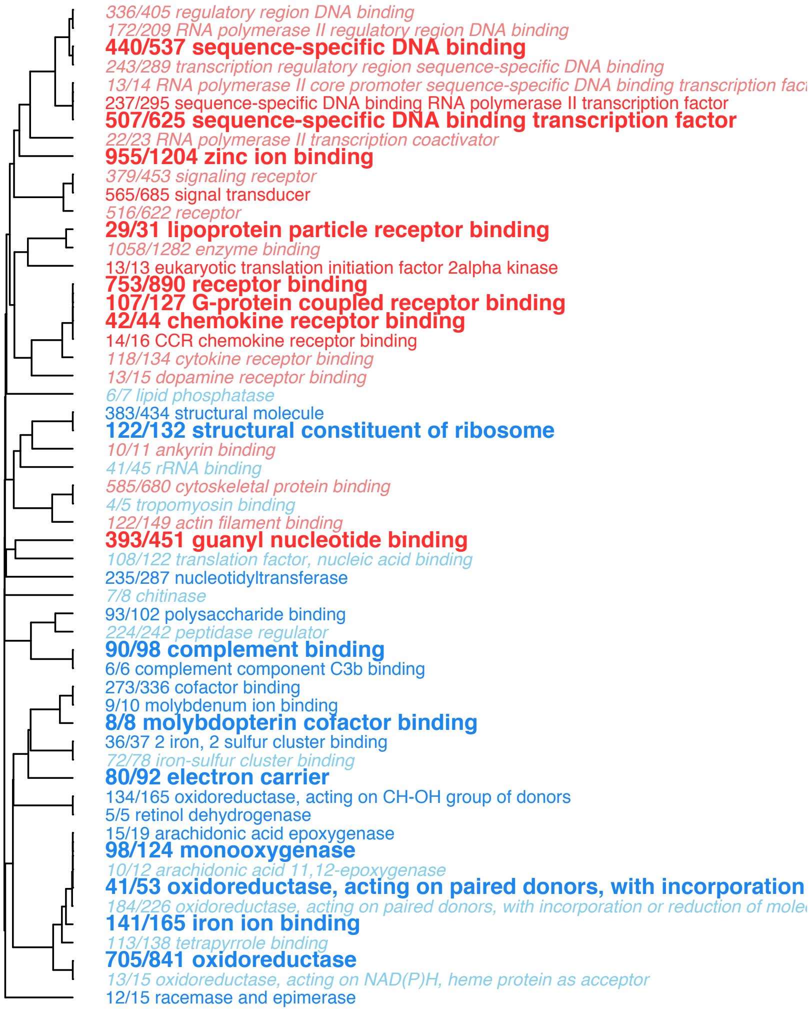

336/405 regulatory region DNA binding
172/209 RNA polymerase II regulatory region DNA binding
440/537 sequence-specific DNA binding
243/289 transcription regulatory region sequence-specific DNA binding
13/14 RNA polymerase II core promoter sequence-specific DNA binding transcription fac
237/295 sequence-specific DNA binding RNA polymerase II transcription factor
507/625 sequence-specific DNA binding transcription factor
22/23 RNA polymerase II transcription coactivator
955/1204 zinc ion binding
379/453 signaling receptor
565/685 signal transducer
516/622 receptor
29/31 lipoprotein particle receptor binding
1058/1282 enzyme binding
13/13 eukaryotic translation initiation factor 2alpha kinase
753/890 receptor binding
107/127 G-protein coupled receptor binding
42/44 chemokine receptor binding
14/16 CCR chemokine receptor binding
118/134 cytokine receptor binding
13/15 dopamine receptor binding
6/7 lipid phosphatase
383/434 structural molecule
122/132 structural constituent of ribosome
10/11 ankyrin binding
41/45 rRNA binding
585/680 cytoskeletal protein binding
4/5 tropomyosin binding
122/149 actin filament binding
393/451 guanyl nucleotide binding
108/122 translation factor, nucleic acid binding
235/287 nucleotidyltransferase
7/8 chitinase
93/102 polysaccharide binding
224/242 peptidase regulator
90/98 complement binding
6/6 complement component C3b binding
273/336 cofactor binding
9/10 molybdenum ion binding
8/8 molybdopterin cofactor binding
36/37 2 iron, 2 sulfur cluster binding
72/78 iron-sulfur cluster binding
80/92 electron carrier
134/165 oxidoreductase, acting on CH-OH group of donors
5/5 retinol dehydrogenase
15/19 arachidonic acid epoxygenase
98/124 monooxygenase
10/12 arachidonic acid 11,12-epoxygenase
41/53 oxidoreductase, acting on paired donors, with incorporation
184/226 oxidoreductase, acting on paired donors, with incorporation or reduction of mole
141/165 iron ion binding
113/138 tetrapyrrole binding
705/841 oxidoreductase
13/15 oxidoreductase, acting on NAD(P)H, heme protein as acceptor
12/15 racemase and epimerase

Figure S10 Gene Ontology (GO) terms significantly enriched with the MWU Test of gene expression for *H. flavolineatum* and *H. macrostomum*, for the categories Biological Process (A), Cellular Component (B) and Molecular Function (C). Size and brightness of GO terms depends on the level of significance, and numbers that precede the category name are the genes present in this particular analysis over the total number of genes that belong to that category. GO terms in red are upregulated categories for *H. flavolineatum*, while blue ones are downregulated.

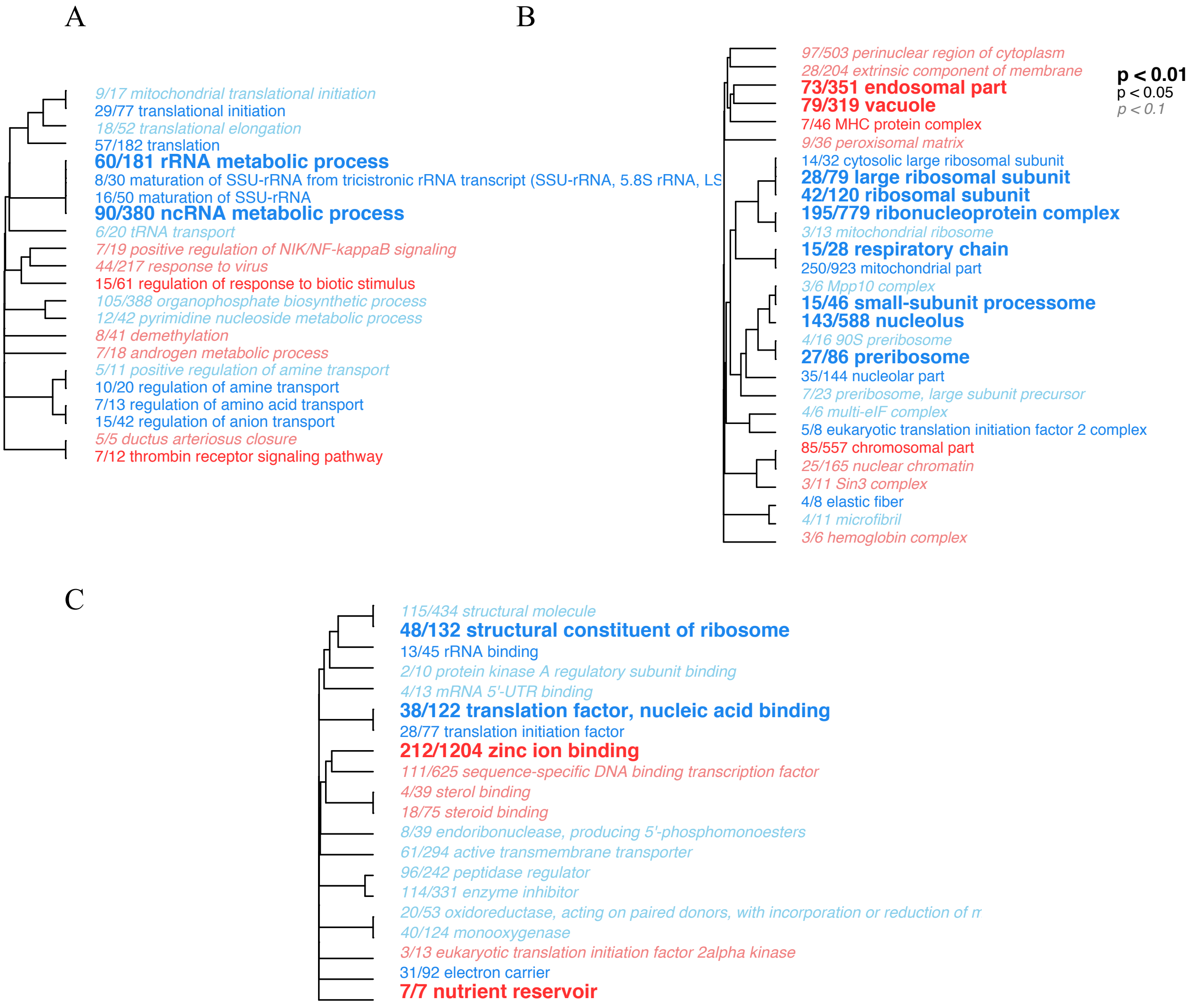

Supplement: Supplemental Information 1 — PDF containing the Supplementary Figs. S1 through S10. [file peerj-07-6541-s001.pdf]
